# m6A eraser FTO modulates autophagy by targeting SQSTM1/P62 in the prevention of canagliflozin against renal fibrosis

**DOI:** 10.3389/fimmu.2022.1094556

**Published:** 2023-01-04

**Authors:** Youjing Yang, Qianmin Li, Yi Ling, Linxin Leng, Yu Ma, Lian Xue, Guoyuan Lu, Yue Ding, Jianzhong Li, Shasha Tao

**Affiliations:** ^1^ Chongqing University Central Hospital and Chongqing Emergency Medical Center, Chongqing, China; ^2^ School of Public Health, Medical College of Soochow University, Suzhou, China; ^3^ Department of Nephrology, The First Affiliated Hospital of Soochow University, Suzhou, Jiangsu, China

**Keywords:** autophagy, canagliflozin, N6-methyladenosine, renal fibrosis, SQSTM1, STAT6

## Abstract

The dysregulation of autophagy contributes to renal fibrosis. N6-Methyladenosine (m6A) RNA modification is a critical mediator of autophagy. Our previous studies have reported that the disorder of the PPARα/fatty acid oxidation (FAO) axis in renal tubular cells is suppressed by STAT6, which is involved in the regulation of renal fibrotic processes. Here, we found that canagliflozin significantly upregulates SQSTM1/P62, promoting PPARα-mediated FAO by inducing autophagy-dependent STAT6 degradation both in TGF-β1-treated HK2 cells and in unilateral ureteral occlusion (UUO) and ischemia–reperfusion (I/R) renal fibrosis mouse models. Knockdown of P62/SQSTM1 led to the impairment autophagic flux and the dysregulation of the STAT6/PPARα axis, which was confirmed by SQSTM1/P62^cKO^ mice with UUO treatment along with bioinformatics analysis. Furthermore, SQSTM1/P62 deficiency in renal tubular cells inhibited canagliflozin’s effects that prevent FAO disorder in renal tubular cells and renal fibrosis. Mechanistically, the level of m6A eraser FTO, which interacted with SQSTM1 mRNA, decreased in the renal tubular cells both *in vitro* and *in vivo* after canagliflozin administration. Decrease in FTO stabilized SQSTM1 mRNA, which induced autophagosome formation. Collectively, this study uncovered a previously unrecognized function of canagliflozin in FTO in the autophagy modulation through the regulation of SQSTM1 mRNA stability in the renal tubular STAT6/PPARα/FAO axis and renal fibrosis.

## 1 Introduction

Renal fibrosis is considered the final common pathogenic process for all kidney diseases. It is characterized by the deposition of the extracellular matrix, tubular atrophy, and loss of peritubular microvasculature (1, 2). When histopathological damage is consistent, structural destruction and functional impairment occur, leading to the poor prognoses of patients with chronic kidney diseases (3). Thus, effective strategies that alleviate renal fibrosis are urgently needed.

Renal tubular cells require high levels of adenosine triphosphate (ATP) through fatty acid oxidation (FAO), and impairment in FAO in these cells causes ATP deficiency, intracellular lipid accumulation, partial epithelial–mesenchymal transition, inflammation, and eventually interstitial fibrosis (4). Our previous study has reported that STAT6, which is activated in the process of kidney fibrosis, negatively regulates PPARα signals. STAT6 inhibition promotes a shift to FAO for energy utilization by inducing PPARα activation in tubular cells, thereby attenuating the lipid accumulation and alleviating renal fibrosis (5). Moreover, STAT6 can be degraded by autophagy-dependent pathways (6). Autophagy is an evolutionarily conserved catabolic pathway that degrades cytoplasmic components, moves organelles, and recycles cytoplasmic contents in eukaryotic cells, contributing to the maintenance of kidney homeostasis and function (7, 8). As one of the most important self-protection mechanisms, basal autophagy in the kidney is critical for maintaining renal homeostasis, structure, and function (9). In acute kidney injury, autophagy is activated as an intrinsic protective mechanism in renal tubular cells (10). However, the role of autophagy in the development of interstitial fibrosis remains a highly controversial issue. On the one hand, autophagy is regarded as an inducer of tubular cell injury and tubular atrophy, leading to fibrosis progression in the kidney. On the other hand, autophagy plays a protective role in renal fibrosis by mediating extracellular matrix protein expression, cell cycle G2/M arrest, or inflammation (11–13). Thus, autophagy is a potential therapeutic target for kidney fibrosis. Moreover, whether autophagy-involved protein degradation is a potential strategy that alleviates renal fibrosis is largely unknown.

Sodium-glucose cotransporter 2 inhibitor (SGLT2i), as a class of anti-hyperglycemic medication, has attracted considerable interest. Canagliflozin (Cana) is the first SGLT2 inhibitor approved by the Food and Drug Administration (FDA). As reported, Cana has been implicated in several biological processes that are independent of SGLT2-involved glucose-lowering effects, including lipid metabolism. Osataphan et al. showed that Cana can promote fatty acid oxidation and ketogenesis in livers (14). Moreover, autophagy can be induced by Cana treatment as reported, but the underlying regulatory mechanisms remain unknown. The most abundant chemical modification of eukaryotic messenger RNA, N6-methyladenosine (m6A) regulates a number of fundamental bioprocesses by targeting the gene expression (15–17). In general, m6A is composed of three kinds of protein, the “Writers,” the “Readers,” and the “Erasers.” Accumulated evidence has shown that m6A modification mediates autophagy (18, 19). However, no evidence of the role of m6A modification in SGLT2i-induced autophagy activation has been obtained.

The aim of this study was to explore the role and modulation of m6A in autophagy regulation toward Cana-mediated renoprotective effects. *In vivo* and *in vitro* evidence has demonstrated that Cana is efficient in providing protection against unilateral ureteral occlusion (UUO)- or ischemia–reperfusion (I/R)-caused renal fibrosis by normalizing the FAO gene program dependent on autophagy. Precisely, the m6A eraser FTO modulates Cana/autophagy by regulating SQSTM1 mRNA stability.

## 2 Materials and methods

### 2.1 Animals and treatments

C57BL/6 mice were purchased from SLAC Laboratory Animal Co., Ltd. Atg7^flox/flox^ mice were gifts from Dr. Jianrong Wang, Medical College of Soochow University (20). SQSTM1^flox/flox^ mice were gifts from Dr. Hongting Zheng, Xinqiao Hospital, Army Medical University (21). All mice were housed with a standard 12-h light/dark cycle, climate-controlled, and pathogen-free facility. All experiments were conducted in accordance with the Guide for the Care and Use of Laboratory Animals, and the study protocols were approved by the Laboratory Animal Welfare and Ethics Committee of Chongqing University. To generate tubular-specific Atg7-deficient and SQSTM1-deficient mice, Atg7^flox/flox^ and SQSTM1^flox/flox^ mice were crossed with γGTcre mice (Cyagen Biosciences). Gender-matched wild-type and specific knockout mice (6–8 weeks old) from the same litter were selected randomly to indicated groups. Two animal studies were performed as follows: a) mice were divided into four groups (n = 8 per group): (i) Ctrl with sham operation, (ii) canagliflozin (Cana) (Selleck S2760; 20 mg/kg in PBS, daily intragastric administration till the mice were harvested), (iii) UUO (sacrificed 7 days after the performance), and (iv) Cana + UUO, and b) mice were divided into four groups (n = 8 per group): (i) Ctrl with sham operation, (ii) canagliflozin (Cana) (Selleck S2760; 20 mg/kg in PBS, daily intragastric administration for consecutive 21 days), (iii) I/R (sacrificed 21 days after the performance), and (iv) Cana + I/R. I/R and UUO operation was performed according to our previous study (5).

### 2.2 Cell culture and treatments

Human proximal renal tubular cell line HK2 was purchased from the Cell Bank of the Chinese Academy of Sciences. HK2 cells were cultured in Dulbecco’s Modified Eagle’s Medium (DMEM, Corning) containing 10% FBS (HyClone) in a 5% CO_2_ incubator at 37°C. TGF-β1 (5 ng/ml) was added to the medium to induce the fibrotic protein expression and lipid accumulation.

### 2.3 Hematoxylin and eosin, immunohistochemical, Oil Red O, and Sirius Red staining

The kidney of each mouse was removed and fixed with 4% paraformaldehyde before embedding. H&E staining was performed for pathological analysis. For IHC staining, kidney sections were incubated with primary antibody LC3 or FTO overnight and then a horseradish peroxidase (HRP) polymer secondary antibody was used. Oil Red O staining and Sirius Red staining were performed using a corresponding kit based on the manufacturer’s instructions (Solarbio, Chondrex). Images were collected and analyzed with a fluorescence microscope (Leica DM 2500).

### 2.4 Assay of serum blood urea nitrogen and lipid parameters

Levels of blood urea nitrogen (BUN) and total cholesterol (TC), low-density lipoprotein cholesterol (LDL), and high-density lipoprotein cholesterol (HDL) in serum were assayed by a corresponding kit (BioAssay Systems, USA, DIUR-500, Nanjing Jiancheng, China, TC: A111-1-1, LDL: A113-1-1, A112-1-1) according to the manufacturer’s construction.

### 2.5 Assay of kidney TG levels

Levels of TG in kidney tissue were measured by using a commercial kit (Nanjing Jiancheng, A:110-1-1). Tissues were weighted and added with PBS (1:9) for homogenization, and the supernatant was obtained. Then, the supernatant and the working solution of the kit were mixed and subjected to measurement of the absorbance at 546 nm. Cellular TG levels were measured using a commercial TG Quantification Colorimetric Kit (BioVision; USA, K622) according to the manufacturer’s protocol. In brief, cells were homogenized in solution containing 5% NP-40 in water and then heated to 100°C. Then, 20 μl cell lysate was adjusted to the volume of 50 μl/well with a triglyceride assay buffer. Finally, the absorbance at 570 nm was measured.

### 2.6 Nucleus and cytoplasm protein isolation

The nucleus and cytoplasm protein of HK2 cells were isolated by using a Nucleus/Cytosolic Extraction Kit (Fude Biological Technology, China, FD0199) based on the manufacturer’s instructions. Briefly, cells were incubated with Nc-Buffer A for 10 min on ice and then added with Nc-Buffer B. After centrifugation, the supernatant was collected to obtain the cytoplasm protein. Nc-Buffer C was added to the deposit to collect the nuclear protein. All the Nc-Buffer should be added with 1% protease inhibitor before use. Nuclear and cytoplasmic proteins were subjected to immunoblot analyses.

### 2.7 Immunoblot analyses

Proteins of cells and tissues were isolated using a RIPA buffer containing protease and phosphatase inhibitor cocktail. Total protein was quantified using the BCA method (Fude Biological Technology, China, FD2001). Equal amounts of protein from each group were separated through an SDS-polyacrylamide gel. The following antibodies were used: STAT6 (sc-374021), p-STAT6 (sc-136019), Arg-1 (sc-166920), TGF-β1 (sc-146), α-SMA (sc-53142), FN (sc-18827), LC3I/II (sc-398822), SQSTM1 (sc-28359), CPT-1α (sc-393070), PPARα (sc-398394), and GAPDH (sc-32233) from Santa Cruz Biotechnology. FTO (AF6936) and N6-Methyladenosine (m6A) Rabbit Polyclonal Antibody (AF7407) were from Beyotime. Protein half-life assays were performed according to our previous protocol (22). Briefly, cell lysates from the control or Cana group were collected at different time points after cycloheximide (CHX) administration and subjected to immunoblot analyses with the anti-STAT6 and anti-GAPDH antibodies. The prestained protein marker (Vazyme Biotech Co., Ltd., MP102-01) was used to identify the specific bands.

### 2.8 Indirect immunofluorescence

For indirect immunofluorescence, cells were fixed on round glass coverslips (Fisher Scientific) with chilled methanol. After incubation with the primary antibodies and the respective secondary antibodies for 50 min each, coverslips were washed with PBS and mounted with antifade mounting solution (Invitrogen). A fluorescence microscope (Leica DM 2500) was used to obtain representative images.

### 2.9 Cell viability assay

Cell viability assay was performed according to the previously described method. Briefly, the potential cytotoxicity of Cana in HK2 cells was assessed by the functional impairment of the mitochondria with 3-(4,5-dimethylthiazol-2-yl)-2,5-diphenyltetrazolium bromide (MTT, Sigma). Approximately 1 × 10^4^ cells per well were plated in a 96-well plate and incubated overnight. The cells were treated with different doses of Cana for 48 h. Then, MTT was added into the cells followed by incubation at 37°C for 2 h. The medium was removed, and 100 μl isopropanol/HCl was added into each well. Absorbance at 570 nm was measured by a synergy 2 multimode microplate reader (BioTek, Seattle, USA).

### 2.10 Actinomycin D treatment

HK2 cells were treated with 5 μg/ml actinomycin D (GC16866, GLPBIO, USA) to inhibit mRNA transcription. Cells were collected at 0, 3, and 6 h. RNA was extracted as below described and used for quantitative polymerase chain reaction (qPCR).

### 2.11 RNA extraction and real-time qPCR

Total RNA was isolated from cells and kidney tissues using TRIzol reagent (CWBiotech, China, CW0580S). Equal amounts of mRNA were synthesized using a iScript cDNA Synthesis Kit based on the manufacturer’s instructions (Vazyme Biotech, China, R333-01) with a 0.2-ml PCR tube (Nest, China, 401001). Real-time PCR was performed using a qPCR SYBR Green Master Mix (Yeasen, 11184). Primer sequences were listed in the supplementary.

### 2.12 RNA immunoprecipitation qPCR

HK2 cells were harvested and lysed in an IP lysis buffer (Beyotime Biotechnology, P0013) containing a protease inhibitor cocktail and RNase inhibitor (Beyotime Biotechnology, R0102-2KU). After centrifugation, the supernatant was collected and 10% of the lysate was taken out for input. The remaining lysate was added to anti-FTO antibody incubated with protein A/G agarose beads at 4°C for 24 h. IgG was used for negative control RNA immunoprecipitation (RIP) reaction. 10% of the complex was collected to test the efficiency of immunoprecipitation after resuspending with a lysis buffer. The remaining lysate was subjected to centrifugation, and RNA was extracted as described above. RNA enrichment was normalized to the input control.

### 2.13 Methylated RNA immunoprecipitation qPCR

Total RNA was extracted as described above. An EpiQuik™ CUT&RUN m6A Enrichment kit (EpiGentek, USA, P-9018) was used to monitor the status of m6A in RNA. 200 ng of RNA was collected and stored at -80°C after cleavage. Anti-m6A antibody or IgG was incubated with affinity beads for 90 min and then washed with a wash buffer. A protein digestion buffer containing proteinase K was added and incubated with an antibody–RNA complex for 15 min at 55°C. Next, the RNA binding beads were resuspended and added to the complex and then the RNA was eluted with an elution buffer. The input of RNA and eluted RNA was subjected to qPCR as described above.

### 2.14 m6A dot blot

Total RNA was isolated as described above and spotted onto nylon membranes. Then, the membranes were crosslinked by ultraviolet (UV) and subjected to methylene blue and then blocked for 1 h with a blocking buffer (5% milk in phosphate-buffered saline with 1% Tween 20). Corresponding secondary antibodies were added and incubated for 1 h.

### 2.15 m6A quantification

The levels of global m6A in mRNA were measured by using an EpiQuik m6A RNA Methylation Quantification kit (Colorimetric) (EpiGentek, Germany) based on the manufacturer’s instruction.

### 2.16 Live-cell imaging

HK2 cells were transfected with mRFP-GFP-LC3 for 24 h and then either left untreated or separately treated with rapamycin, CQ, or Cana. Finally, cells were gently washed with PBS and images were captured with a fluorescence microscope (Leica DM2500, USA).

### 2.17 Microarray data processing

The expression profile and corresponding annotation platform of GSE118337 were downloaded from the National Center for Biotechnology Information (https://www.ncbi.nlm.nih.gov/) (23). The profile was then normalized by function *normalizeBetweenArrays()*. The normalized microarray series matrix was processed by the limma R package (24) to calculate the log fold change, p value, and adjusted p value of gene expression for further analysis, and genes that meet |log2FC| >1 and p < 0.05 were considered differentially expressed. A volcano plot and heatmap created by R were graphed to show the overall condition of the data.

### 2.18 Protein–protein interaction analysis

Genes in GSE118337 regulated by TGF-β1 and restored by Cana (p < 0.05, |log2FC| >1) were used to create a protein–protein interaction (PPI) network on String and visualized by Cytoscape (25, 26). Retrieved from functional enrichment results, the genes related to fibrosis and fatty acid oxidation were highlighted.

### 2.19 Enrichment analysis

The upregulated and downregulated genes in GSE118337 were respectively used for biological process functional enrichment on Metascape (27), the results of which were used to create bar plots showing functions related to the present investigation by R. Gene sets used for functional enrichment analysis and gene set enrichment analysis (GSEA) were downloaded from GSEA (http://www.gsea-msigdb.org/gsea/index.jsp) (28, 29). Then, software GSEA 4.2.2 and R package “ClusterProfiler” were employed for GSEA and visualization (30).

### 2.20 Correlation analysis

Correlation between the expression level of genes was calculated by Pearson correlation analysis. The p-value of the correlation coefficient was analyzed with package “hmisc,” and p-value <0.05 was considered statistically significant. An absolute value of Pearson correlation coefficients between 0.6 and 1 indicated a strong correlation, and that between 0.4 and 0.6 indicated a moderate one. The correlation was finally visualized by software Cytoscape and R package “corrplot” (31).

### 2.21 Construction of the autophagy gene signature

We applied a methodology to quantify the autophagy level (autophagy score) of samples. First, we chose 19 genes with frequent existence in pathways related to autophagy or reported in previous studies (32). Next, principal component analysis (PCA) was performed to establish the autophagy gene signature of genes selected above. Finally, we selected principal components 1 as signature scores.

### 2.22 Statistical analysis

The investigators were blinded to group allocation. Data were presented as mean ± SD. GraphPad Prism 8.0 and R statistical software were performed for graphics and statistical analyses. Unpaired Student’s t tests were applied for comparison between two groups. For multiple comparison analyses, one-way ANOVA with Bonferroni’s correction were performed. The differences with *p < 0.05 was considered statistically significant.

## 3 Results

### 3.1 Cana showed tight regulation both on renal fibrosis and fatty acid metabolism

**Figure 1:**
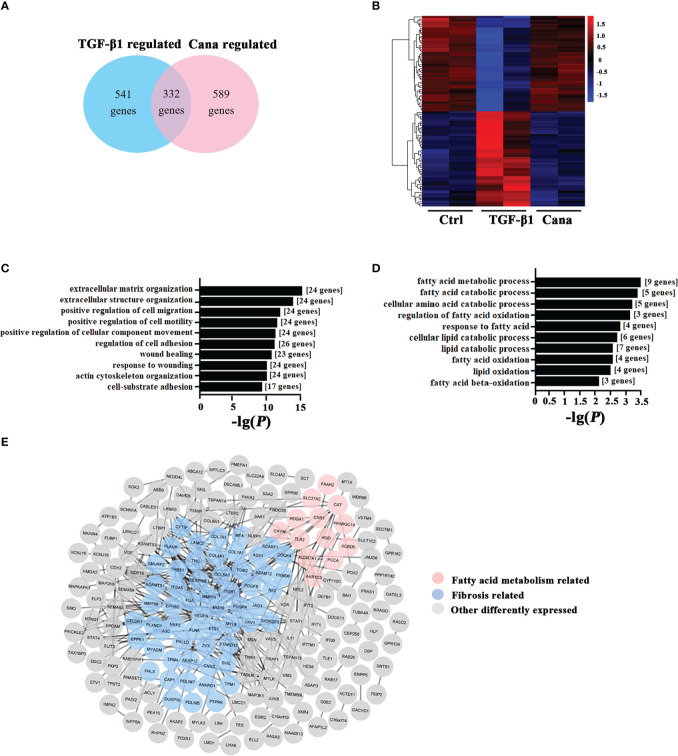
Cana positively regulated the abnormality of lipid metabolism and tubulointerstitial fibrosis. **(A–E)** The microarray expression dataset GSE118337 was retrieved from the GEO database. **(A)** A total of 541 genes were regulated by TGF-β1, and 589 genes were by Cana (*p* < 0.05, |log2FC| >1), with 332 genes at their intersection. **(B)** The heatmap displayed the top 50 upregulated and downregulated genes of two samples in the TGF-β1 group and two samples in the Cana group by z-score, in comparison with the Ctrl group. GO analysis was separately conducted with genes upregulated by TGF-β1 and downregulated by Cana **(C)** and genes downregulated by TGF-β1 and upregulated by Cana **(D)**, and the 10 highest-ranking biological process terms are shown. **(E)** The interaction network of above 332 genes both regulated by TGF-β1 and Cana, in which fibrosis-related genes and fatty acid metabolism-related genes according to enrichment results are highlighted.

To verify the effects of Cana on renal fibrosis and lipid metabolism, the microarray expression dataset GSE118337 retrieved from the GEO database was analyzed. As shown in **Figure 1A**, there were 541 genes regulated by TGF-β1 and 589 genes regulated by Cana (*p* < 0.05, |log2FC| >1), with 332 genes at their intersection. 61.3% of the genes changed by TGF-β1 were further restored by Cana, revealing the potential therapeutic effect of Cana on renal fibrosis (**Figure 1B**). Among them, 193 genes and 139 genes were separately upregulated and downregulated by TGF-β1 and restored by Cana. Enrichment functions of all genes regulated by both TGF-β1 and Cana (*p* < 0.05) were conducted by Metascape (https://metascape.org/). As shown in **Figures 1C–E**, gene ontology terms associated with fibrosis and fatty acid metabolism were significantly mediated and the PPI network was graphed, suggesting that fatty acid metabolism and fibrosis-related genes are closely interacted with each other. These data showed that Cana exhibited the significant modulation both on renal fibrosis and fatty acid metabolism.

### 3.2 Cana improved aberrant lipid metabolism and renal fibrosis induced by UUO and I/R

**Figure 2:**
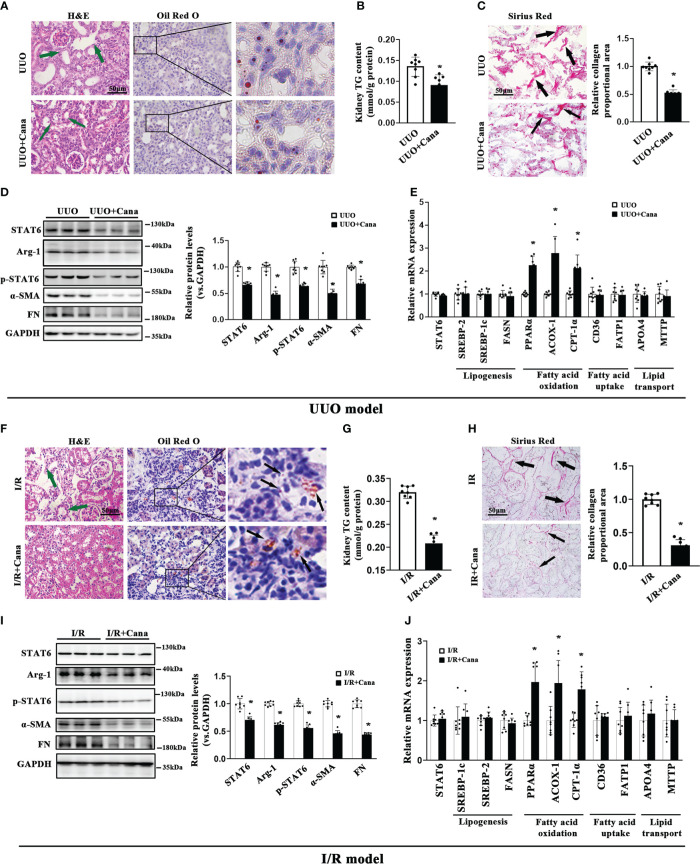
Cana intervention attenuated aberrant lipid metabolism and tubulointerstitial fibrosis in UUO or I/R mice. A tubulointerstitial fibrosis model was conducted by UUO or I/R operation. The mice of UUO or UUO+Cana were sacrificed at day 7, and mice of I/R or I/R+Cana were sacrificed at day 21. **(A)** Representative micrographs for H&E and Oil Red O staining in the kidney sections from the indicated groups. **(B)** TG content was determined in the kidneys from the indicated groups. **(C)** Representative micrographs for Sirius Red staining and quantification of relative collagen proportion in the kidneys from the indicated groups. **(D)** Kidney tissue lysates from each group were subjected to immunoblot analyses with the indicated antibodies. Representative blots of three independent samples in each group were shown, and quantification of relative protein expression was determined. **(E)** The mRNA levels of genes related to lipid metabolism in the kidneys from the indicated groups. Mice received intragastric administration of saline or Cana intervention after IR operation. **(F)** H&E and Oil Red O staining, **(G)** TG content, **(H)** Sirius Red staining, **(I)** immunoblot analyses, and **(J)** qPCR. Results were expressed as the mean ± SD (n = 8, *p < 0.05, UUO *vs.* UUO + Cana or IR *vs.* IR + Cana).

Based on the bioinformatic analysis above, we next conducted an *in vivo* study *via* establishing UUO and I/R models to confirm the protection of Cana on renal fibrosis. H&E staining showed that the histological injury caused by UUO and I/R was attenuated by Cana, so did the lipid accumulation as shown by Oil Red O staining and triglyceride (TG) content in the kidney, without damage on renal function (**Figures 2A, B, F, G**, **S1A**). In addition, Sirius Red staining exhibited the alleviated collagen deposition by Cana (**Figures 2C, H**). Our previous study showed that STAT6, a key nuclear transcription factor, is activated in fibrosis-associated tissue disorder and it could negatively regulate PPARα related-lipid metabolism, which contributes to lipid accumulation in renal tubular cells and kidney fibrosis (5). To explore the potential mechanism of Cana on the regulation of FAO and renal fibrosis, the expressions of STAT6 signal-related protein (STAT6, p-STAT6, Arg-1) and fibrosis-related protein (α-SMA, FN) were detected by immunoblot analyses (**Figures 2D, I**). Interestingly, here we found that STAT6 was suppressed in the kidney of UUO or I/R mice with Cana treatment. The deactivation of the STAT6 pathway was accompanied by reduced expressions of fibrosis-related protein α-SMA and FN in UUO and I/R kidney. Consistently, Cana mainly upregulated the FAO in the kidney with UUO and I/R performance (**Figures 2E, J**). These data suggested that Cana may attenuate UUO- and I/R-induced renal fibrosis through STAT6 inhibition and FAO activation.

### 3.3 Cana alleviated TGF-β1-induced HK2 cell aberrant lipid metabolism and fibrosis-related protein expression through suppressing STAT6 expression

**Figure 3:**
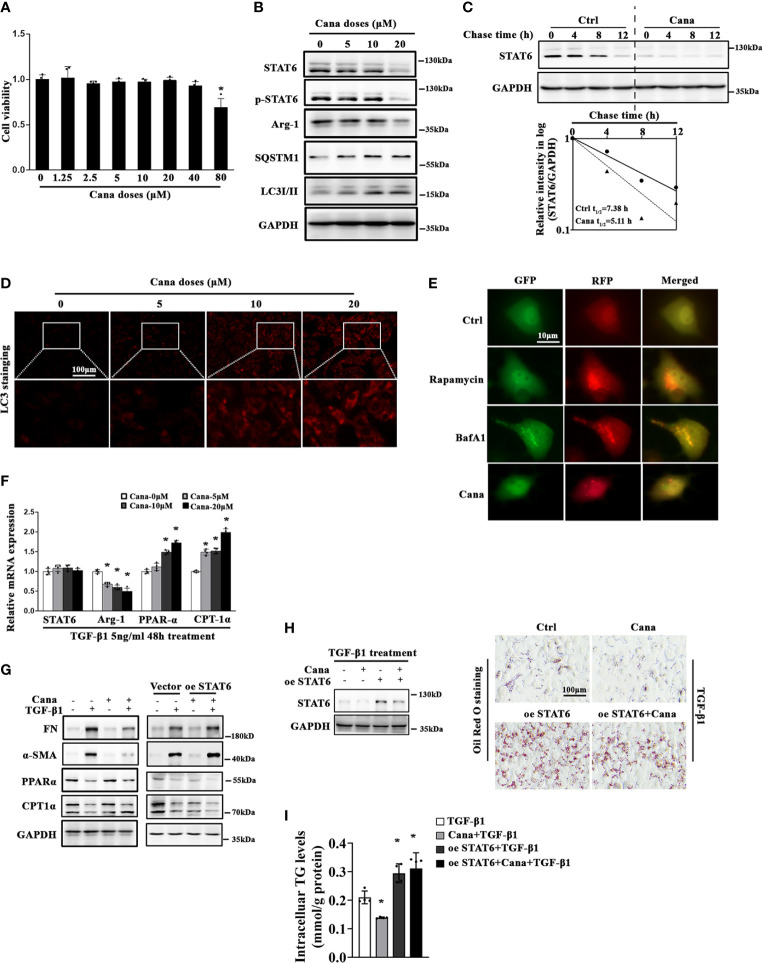
Cana causes STAT6 degradation through inducing autophagy. **(A)** HK2 cells were treated with the indicated doses of Cana for 48 h serum-free medium culture and cell viability was determined using MTT assay. (n = 4, *p < 0.05, Cana 0 μM *vs.* other doses of Cana). **(B)** HK2 cells were treated with Cana (0–20 μM) for 24 h, and then cell lysates were harvested and subjected to immunoblot analyses with the indicated antibodies. **(C)** To test its protein stability, the half-life of STAT6 was determined. HK2 cells were left untreated or treated with Cana (20 μM) for 24 h, and CHX (50 μM) was added at different time points. The intensity of STAT6 and GAPDH bands was quantified and plotted against the time after CHX addition. **(D)** HK2 cells were treated with Cana (0–20 μM) for 24 h and subjected to immunofluorescence staining of LC3. **(E)** HK2 cells were transfected with a tandem mRFP-GFP-LC3 construct for 24 h and then either left untreated or separately treated with rapamycin (1 μM), BafA1 (100 nM), or Cana (40 μM) for 24 h. Representative micrographs of the indicated cells are shown. **(F)** The mRNA levels of STAT6, Arg-1, PPARα, and CPT-1α in HK2 cells treated with Cana (0–20 μM) for 24 h were measured by qPCR. (*p < 0.05, Cana 0 μM *vs*. other doses of Cana). **(G–I)** HK2 cells were transfected with vector or plasmid of STAT6 overexpression. Followed by 24 h serum-free medium culture, cells were treated with TGF-β1 (5 ng/ml) for another 24 h and cotreated with or without Cana (20 μM). **(G)** Immunoblot analysis was conducted with the indicated antibodies. **(H)** Immunoblot analyses were employed to confirm the efficiency of overexpressing STAT6. Representative microphages showed the Oil Red O staining in HK2 cells with the indicated treatments. **(I)** TG content was determined enzymatically. (n = 4, *p < 0.05, TGF-β1 *vs.* TGF-β1 with other treatments).

Next, we conducted *in vitro* studies using HK2 cells to further confirm Cana’s protection effects observed in animal studies above. MTT assay was employed to select the dose range (0–40 μM) in the following *in vitro* experiments (**Figure 3A**). Consistently, Western blot showed that Cana decreased the expression and activation of STAT6 signals (STAT6, p-STAT6, and Arg-1) in a dose-dependent manner along with the increased expressions of SQSTM1 and LC3II and less effect on LC3I (**Figure 3B**). Since STAT6 could be degraded by autophagy according to others’ and our previous studies, the stability of STAT6 was next detected. Cana shortened STAT6 half-life from 7.38 to 5.11 h (**Figure 3C**). Consistent with **Figure 3B**, immunofluorescence (IF) staining showed that Cana increased LC3 expression in a dose-dependent manner (**Figure 3D**). Moreover, a tandem mouse red fluorescent protein (mRFP)-GFP-LC3 construct was employed. Two positive controls were conducted to differentiate increased formation of autophagosomes (rapamycin) from blockage of autophagosome degradation (bafilomycin A1, BafA1). As shown in **Figure 3E**, cells treated with Cana contained red puncta but less yellow puncta, which was the same as rapamycin, indicating that Cana may act as an inducer of autophagy. Moreover, qPCR and Western blot assays indicated that Cana alleviated TGF-β1-induced abnormal lipid metabolism and fibrotic protein expression (**Figures 3F, G**). Then, the effects of downregulated STAT6 by Cana were further explored. Overexpression of STAT6 exacerbated TGF-β1-induced aberrant lipid metabolism and fibrotic protein expression and impaired the protective effect of Cana, which further confirmed that Cana improved FAO and fibrosis *via* autophagy degradation of STAT6 (**Figures 3G–I**). These data suggested that Cana suppressed STAT6 through activation of autophagy to alleviate TGF-β1-induced aberrant lipid metabolism and fibrotic protein expression.

### 3.4 Autophagy mediated Cana’s protection effects for renal fibrosis *via* inducing STAT6 degradation in tubular cells

**Figure 4:**
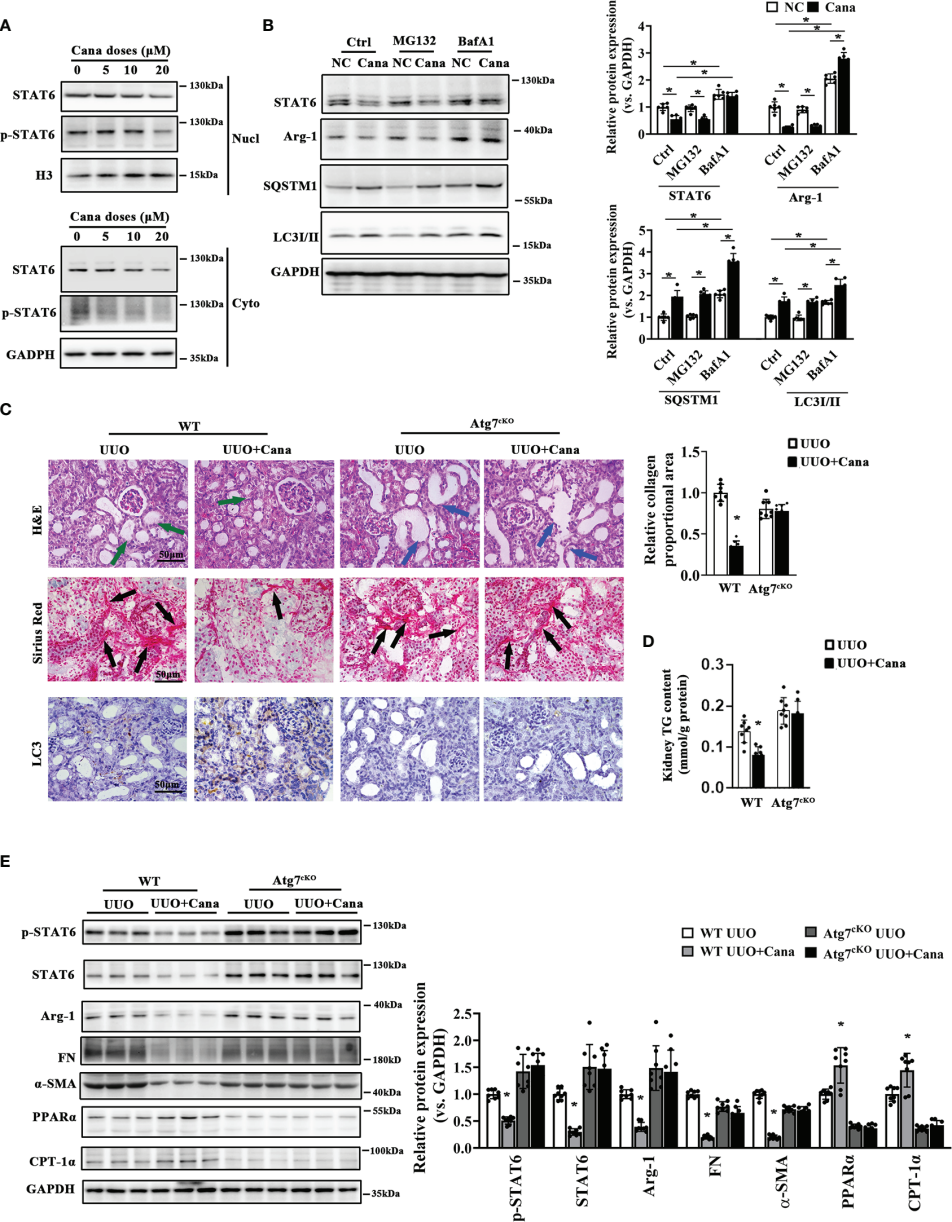
Atg7 deficiency vanished the protection of Cana in tubulointerstitial fibrosis. **(A)** HK2 cells were treated with Cana (0–20 μM) for 24 h. The protein expression of STAT6 and p-STAT6 in nucleus and cytoplasm was separated and determined by immunoblot analyses. **(B)** HK2 cells were pretreated with MG132 (10 μM) or BafA1 (100 nM) for 4 h and cotreated with or without Cana after being cultured with serum-free medium for 24 h. The cell lysates were subjected to immunoblot analyses with the indicated antibodies. **(C–E)** WT and Atg7^cKO^ mice received intragastric administration of saline or Cana (20 mg/kg) after UUO operation. **(C)** Representative images of H&E, Sirius Red, and IHC staining of kidney sections from the indicated group of mice. Quantification of the relative collagen proportional area from each group of mice was performed. **(D)** TG content in kidney tissue was measured by a commercial kit. **(E)** Immunoblot analyses for the protein levels of p-STAT6, STAT6, Arg-1, FN, α-SMA, PPARα, CPT-1α, and GAPDH in kidney from the indicated group with quantification on the right panel (n = 8, *p < 0.05, UUO *vs*. UUO + Cana).

Since we found that Cana decreased STAT6, HK2 cells treated with different doses of Cana were separated to detect STAT6 expression in the cytoplasm and nucleus, respectively. The results showed that the expression of STAT6 in both cytoplasm and nucleus was decreased after Cana treatment (**Figure 4A**). To further determine the method of STAT6 degradation, cells were treated with Cana along with proteasome inhibitor (MG132) or autophagy inhibitor (BafA1). Results showed that the expressions of SQSTM1 and LC3II were both increased after Cana treatment. Higher levels of LC3II and SQSTM1 were found in the Cana and BafA1 combined treatment group (**Figure 4B**), which was consistent with **Figure 3E** that Cana induced the autophagosome formation. Moreover, only BafA1 treatment reversed the decrease in STAT6 signaling.

Whether autophagy is involved in the protective effect of Cana against tubulointerstitial fibrosis *in vivo* was further confirmed. There was no obvious different histological change and collagen deposition (**Figure S2A**). Interestingly, remarkable tubular atrophy and interstitial collagen deposition were observed in the WT group with UUO performance, which was significantly ameliorated by Cana administration. These protective effects of Cana were inhibited in the tubular-Atg7^-/-^ (Atg7^cKO^) group (**Figures 4C, D**). Consistently, Western blot showed that Atg7 deficiency in tubular cells vanished Cana’s effect of suppressing STAT6 signaling, inhibiting fibrotic protein expression, and increasing FAO-related protein expression (**Figure 4E**). These data suggested that Cana improved renal tubular cells’ FAO and kidney fibrosis through degradation of STAT6 in an autophagy-dependent manner.

### 3.5 Cana-induced autophagy was significantly associated with SQSTM1

**Figure 5:**
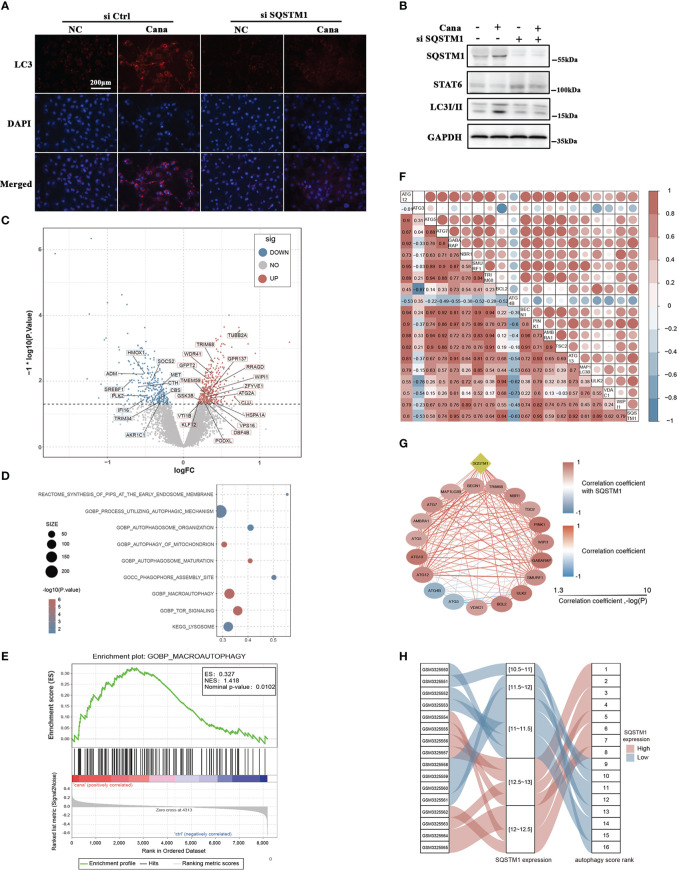
Autophagy was significantly associated with SQSTM1/P62 and Cana. **(A, B)** HK2 cells were transfected with or without SQSTM1 siRNA. Followed by 24 h serum-free medium culture, cells were treated with or without Cana (20 μM) for another 24 h. **(A)** Representative microphages of immunofluorescence from the indicated group are shown. **(B)** Immunoblot analyses was performed. **(C)** Volcano map showing that DEGs between Cana treated and control samples with autophagy-related genes were labeled. **(D)** Functional enrichment analysis of DEGs. **(E)** Gene set enrichment analysis result. **(F, G)** Correlation between SQSTM1 and genes related to autophagy. **(H)** The variation of SQSTM1 expression level and its corresponding autophagy score rank shown by alluvial diagram.

SQSTM1 is well known as the receptor of autophagy-related degradation. In the above experiments, we found that SQSTM1 was induced after Cana treatment (**Figures 3B**, **4B**). To elucidate the functional role of SQSTM1, a siRNA-based strategy was used to interfere HK2 cells’ SQSTM1 expression and then detected the change of STAT6 and autophagy levels. The efficiency of siSQSTM1 was confirmed at the protein level. During knockdown of SQSTM1, the suppressed expression of STAT6 was alleviated while the increased expression of LC3II caused by Cana was inhibited (**Figure 5B**). In line with the results from Western blot, IF staining also showed that Cana barely induced LC3 expression with siSQSTM1 transfection (**Figure 5A**).

In order to further identify that Cana and SQSTM1 were related to autophagy, we systematically searched publicly available renal gene-expression data sets and chose GSE118337 for the next analysis. The Cana group and control group were selected as the object (33). Differentially expressed genes (DEGs) between the two groups were determined using the R package limma. The results represented by volcano plot showed that many DEGs were related to autophagy (**Figure 5C**). Moreover, functional enrichment analysis revealed that DEGs were significantly enriched in pathways related to autophagy (**Figure 5D**). To further explore the changes of autophagy, gene set enrichment analysis (GSEA) was used to observe the enrichment of gene signatures in different phenotypes. The results showed that autophagy-related genes were significantly enriched in the Cana-treated group (**Figure 5E**). It was concluded that Cana treatment was closely related to autophagy of renal proximal convoluted tubule epithelial cells and might upregulate the level of autophagy. Additionally, the correlation between SQSTM1 and 19 feature genes related to autophagy was calculated. The results indicated that autophagy-related genes were mostly positively correlated with SQSTM1 (**Figures 5F, G**). Then, 19 feature genes were extracted for further PCA to establish the autophagy score and rank. Samples were ranked based on gene expression of SQSTM1, and its relationship with PCA score was visualized with an alluvial diagram which showed that a high SQSTM1 expression got a high autophagy score rank and vice versa (**Figure 5H**). The above results indicated that there was a close relationship among autophagy, Cana treatment, and SQSTM1.

### 3.6 Cana enhanced the stability of SQSTM1 mRNA through inhibiting FTO

**Figure 6:**
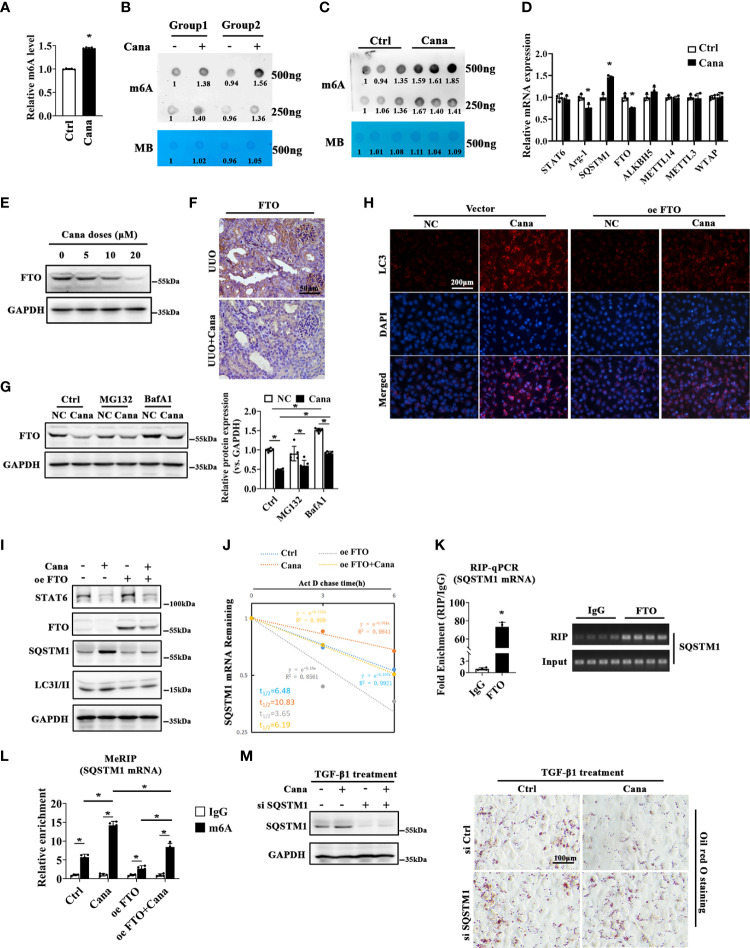
FTO was downregulated by Cana to enhance the stability of SQSTM1 mRNA through installing m6A modification of its mRNA. m6A levels of HK2 cells treated with or without Cana (20 μM) were detected by using the EpiQuik™ m6A RNA methylation quantification kit **(A)** and RNA dot blot analyses **(B)**. Methylation blue (MB) staining served as a loading control. **(C)** RNA dot blot analyses of m6A levels in mice from the indicated group. **(D)** HK2 cells treated with or without Cana (20 μM) were harvested and subjected to qPCR to determined STAT6, Arg-1, SQSTM1, FTO, ALKBH5, METTL14, METTL3, and WTAP mRNA levels. (n = 4, *p < 0.05, Ctrl *vs.* Cana). **(E)** HK2 cells treated with Cana (0–20 μM) for 24 h were subjected to detected the protein levels of FTO by immunoblot analyses. **(F)** Mice received intragastric administration of saline or Cana (20 mg/kg) after UUO treatment. The expression of FTO was determined through IHC staining. **(G)** HK2 cells were pretreated with MG132 (10 μM) or BafA1 (100 nM) for 4 h and cultured with serum-free medium for 24 h. Then, the cells were left and treated with or without Cana (20 μM) for another 24 h. Immunoblot analyses was performed to detect the protein level of FTO. **(H–J, L)** HK2 cells were transfected with or without plasmid of FTO overexpression 48 h. Then, cells were treated with or without Cana (20 μM) for another 24 h. **(H)** The levels of LC3 in HK2 cells from the indicated group were detected through immunofluorescence staining. **(I)** Immunoblot analysis was performed to determine the protein levels of STAT6, FTO, SQSTM1, LC3I/II, and GAPDH. **(J)** qPCR analyses of SQSTM1 mRNA stability in HK2 cells from the indicated group (*p < 0.05). **(K)** RNA immunoprecipitation (RIP)-qPCR analyses revealed the SQSTM1 mRNA level enriched by the FTO antibody. The agarose gel electrophoresis analyses of qPCR products are shown on the panel (*p < 0.05). **(L)** MeRIP-qPCR assay indicated the m6A modification level of HK2 cells from the indicated group (*p < 0.05). **(M)** HK2 cells transfected with or without SQSTM1 siRNA for 24 h and then treated with Cana (20 μM) along with TGF-β1 (5 ng/ml) for another 48 h. The efficiency of knockdown SQSTM1 in HK2 cells was confirmed by immunoblot analyses. Representative microphages showed the Oil Red O staining in HK2 cells with indicated treatments.

In the above results, we found that SQSTM1 is a key factor in the protection of Cana on renal fibrosis; we next sought to analyze its potential mechanism. To confirm Cana’s function from the perspective of m6A modification, we firstly identified whether m6A participates in the process of Cana administration. It was found that m6A levels were significantly increased in HK2 cells following Cana administration (**Figures 6A, B**). Consistently, the kidney of mice treated with Cana exhibited the same trend (**Figure 6C**). It was reported that the m6A level was mainly modified by methyltransferase or demethylase. Thus, we examined related gene expression to verify whether Cana regulates through mediating RNA methyltransferases or demethylases. As shown in **Figure 6D**, the expression of SQSTM1 was consistently upregulated and Arg-1 (a typical STAT6 downstream gene) expression was decreased. Notably, Cana significantly inhibited FTO expression, with no obvious change of other methyltransferase- or demethylase-related genes. In accordance with the results of qPCR, we validated that Cana deceased FTO at the protein level both *in vitro* and *in vivo* (**Figures 6E, F**). Next, the transcriptional downregulation manner of FTO by Cana was further confirmed in the cells with either proteasome inhibitor (MG132) or autophagy blocker (BafA1) treatment. Interestingly, the levels of FTO in the BafA1-treated group were dramatically increased compared with the Ctrl group. However, BafA1 treatment could not recover the decrease of FTO caused by Cana, as well as MG132, which indicated that the downregulation of FTO by Cana is not caused by increased protein degradation through either proteasome or autophagy (**Figure 6G**). Next, IF staining and Western blot were employed to further investigate the effect of FTO on autophagy. The results showed that the increased LC3II and SQSTM1 was inversed with FTO overexpression (**Figures 6H, I**). Also, a tandem mouse red fluorescent protein (mRFP)-GFP-LC3 construct was employed. As shown in **Figure S3A**, HK2 cells treated with Cana contained red puncta but less yellow puncta, indicating its induction of autophagy. However, overexpression of FTO attenuated Cana’s effect on autophagy induction. Moreover, we found that Cana treatment increased the stability of SQSTM1 mRNA, whereas overexpressing FTO resulted in decreased stability of SQSTM1 mRNA. These data suggested that Cana decreased FTO transcription, which negatively regulated autophagy through modulation of the SQSTM1 mRNA stability (**Figure 6J**).

Then, we profiled that SQSTM1 mRNA contained the potential m6A modification region (position: 1906) with an FTO-binding motif as shown in **Figure S4**. Next, the RNA immunoprecipitation (RIP)-qPCR experiment was conducted and the results confirmed that FTO protein interacted with SQSTM1 mRNA (**Figure 6K**). Moreover, the methylated RNA immunoprecipitation (MeRIP) assay showed that Cana increased the m6A modification of SQSTM1 mRNA, whereas overexpression of FTO attenuated Cana’s effect of increasing SQSTM1 mRNA m6A modification (**Figure 6L**). With the vital role of SQSTM1 in the modulation of autophagy, we then downregulated SQSTM1 to observe the effect of Cana. As shown in **Figure 6M**, the knockdown efficiency was confirmed using immunoblot analysis. Cana significantly alleviated TGF-β1-induced lipid accumulation. Oppositely, siSQSTM1 exacerbated TGF-β1-induced lipid accumulation and impaired the protective effect of Cana. Collectively, these data suggested that Cana showed negative regulation on FTO and enhanced SQSTM1 mRNA stability.

### 3.7 SQSTM1 deficiency eliminated the protection effect of Cana against renal fibrosis

**Figure 7:**
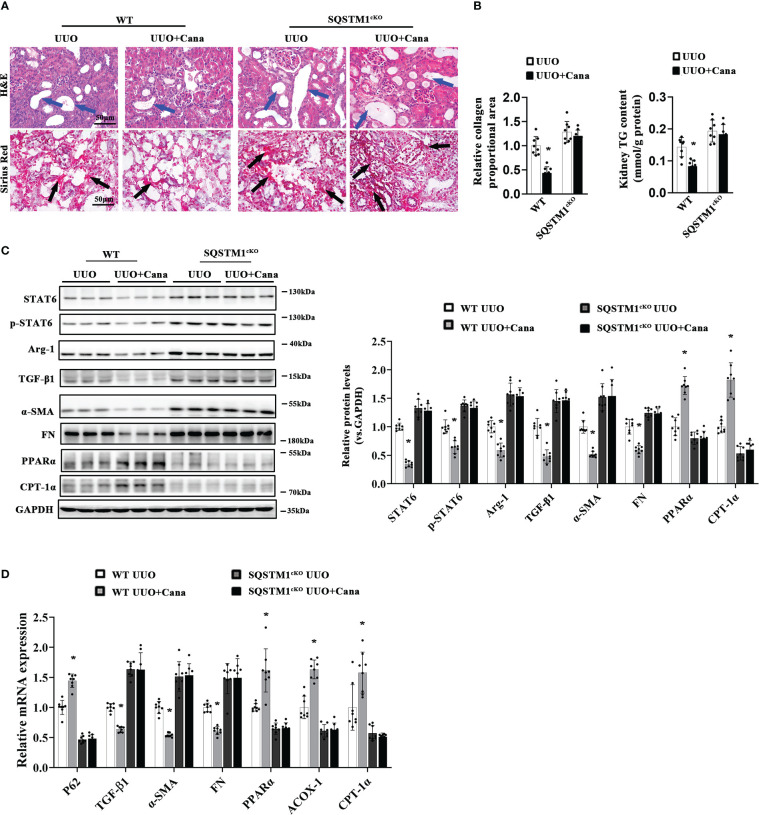
SQSTM1 deletion eliminated the protection effect of Cana against tubulointerstitial fibrosis. **(A)** Representative micrographs for H&E and Sirius Red staining of kidney sections from the indicated groups of mice. Quantification of the relative collagen proportional area from each group of mice was performed. **(B)** TG content was determined in the kidneys from each group of mice. **(C)** Immunoblot analyses in the kidneys from each indicated group with quantification. **(D)** The mRNA levels were measured by qPCR assay in the kidneys from each indicated group. Results are expressed as mean ± SD (n = 8 *p < 0.05, UUO *vs*. UUO+ Cana).

To further provide evidence for the *in vivo* role of SQSTM1, renal tubular-specific SQSTM1 deficiency mice were generated using SQSTM1^flox/flox^ and γGT^Cre^ mice. There were no obvious differences in histological change and collagen deposition, while there were comparisons between WT and SQSTM1^cKO^ mice at 2 months after birth (**Figure S5A**). As shown in **Figure 7A**, obvious tubular atrophy and interstitial collagen deposition were presented in the WT group with UUO treatment, which was ameliorated with Cana administration. These protective effects were dampened in the SQSTM1^cKO^ group. Additionally, SQSTM1^cKO^ mice exhibited more aggravated renal morphological damage and fibrosis in comparison with the indicated WT mice. Also, Cana was found to decrease the TG content in WT mice, whereas there was no change in SQSTM1^cKO^ mice. Furthermore, TG content in SQSTM1^cKO^ mice was even higher than that in WT mice (**Figure 7B**). Consistently, immunoblot and qPCR analyses also showed that suppression of the STAT6 signaling pathway by Cana only in WT mice and the SQSTM1^cKO^ group showed higher levels of STAT6 and p-STAT6 expression compared with the WT group. Consistently, Cana eliminated the alteration of UUO-induced fibrosis-related protein and PPARα-related FAO gene expression, whereas SQSTM1^cKO^ mice exhibited more fibrosis-related gene expression and less FAO-related gene expression in comparison with WT mice (**Figures 7C, D**). These data suggested that SQSTM1 was essential for the function of Cana on renal fibrosis and SQSTM1 deletion eliminated the protection of Cana against renal fibrosis.

## 4 Discussion

Here, our study firstly revealed that Cana ameliorated UUO- or I/R-induced renal lipid metabolism disorder and fibrosis based on the degradation of STAT6 in a SQSTM1/autophagy-dependent manner. Specifically, after Cana administration, relative m6A levels increased *in vivo* and *in vitro*. Cana decreased the expression of m6A eraser FTO and recovered SQSTM1 mRNA stability. Deficiency in endogenous SQSTM1 in mice deteriorated the protective effect of Cana. Overall, our study presented a novel mechanism by which Cana (a commercial antidiabetic drug based on the inhibition of SGLT2) acts to counter to abnormal renal fatty acid metabolism and interstitial fibrosis through the m6A-modified SQSTM1/autophagy/STAT6 axis.

Renal fibrosis is a complex and coordinated process, which involves multiple cell types and cytokines (34–36). Tubular cells are highly specialized cells that use high amounts of ATP to maintain intense reabsorption and excretion processes (37). In tubular cells, FAO impaired by a specific inhibitor or endogenic gene knockdown can induce aberrant lipid accumulation, resulting in the progression of kidney fibrosis (38). STAT6 is a major regulatory transcription factor for type II-related gene expression (involving TGF-β), and our previous study revealed the crucial role of STAT6 in regulating PPARα-mediated FAO in tubular cells and kidney interstitial fibrosis (5, 39, 40). Substantial evidence indicates that SGLT2 inhibitors elicit unanticipated renoprotective effects in non-diabetic and diabetic kidney disease (41, 42). Thus, improving improper lipid metabolism reprogramming represents a pivotal target by which SGLT2i mitigates kidney fibrosis after renal damage, but the mechanism by which they improve lipid metabolism is elusive. In our study, the potential roles of Cana in regulating fibrosis and FAO were firstly verified and evaluated by relative bioinformatics analysis (**Figure 1**). *In vivo* studies were also carried out to demonstrate that Cana can improve UUO- or I/R-induced renal fibrosis and FAO dysregulation. The induction of STAT6 in fibrotic kidneys was blunted by Cana treatment (**Figure 2**). Glycolysis is critical for energy metabolism, and SGLT2 inhibition can suppress fibrogenesis in diabetic kidneys by suppressing aberrant glycolysis (43). Moreover, Dufort et al. found that STAT6 mediated glycolysis in B cells (44). Therefore, STAT6/glycolysis may be one of the mechanisms underlying the protective effects of Cana and warrants further investigation.

Next, the mechanisms about Cana-suppressed STAT6 induction were explored, which is important for intracellular homeostasis. Autophagy is a well-known major intracellular degradation system where cytoplasmic materials are delivered and degraded by lysosome, and the process can be activated by many stimuli including starvation, genotoxicity, and inflammatory stress (45–48). As we previously reported, STAT6 can be degraded *via* the autophagosomal pathway (22). Moreover, autophagy can be induced by SGLT2i through several signaling pathways, such as AMPK/mTOR and SIRT1 or SIRT3 signaling (49–52). Consistently, the results of our bioinformatic analysis here also indicated the close association between autophagy and Cana treatment (**Figure 4**). Thus, the renoprotective effect exerted by Cana treatment was speculated through autophagy-mediated STAT6 degradation, and this prediction was confirmed by the obtained results. First, both *in vivo* and *vitro* results showed the induction of autophagy following Cana treatment, and STAT6 protein degradation was mostly affected by the autophagolysosomal pathway, rather than the ubiquitin–proteasomal pathway. Second, the inhibition of autophagy impaired the protection of Cana on lipid metabolism disorder and renal fibrosis by Atg7 knockout in tubular cells.

In the current study, SQSTM1 was upregulated dose-dependently by Cana treatment in HK-2 cells, hinting that SQSTM1 is a potential target gene of Cana. During the process of autophagy, SQSTM1/P62 acts as a vital and multifunction protein. In general, SQSTM1 is a robust indicator of dynamic autophagic flux, given that it is an autophagy receptor that directly interacts with selected cargoes for degradation. Primarily, the inhibition of autophagy leads to intracellular SQSTM1 accumulation (53). Moreover, SQSTM1 is an upstream regulator of autophagy and binds to arginylated substrates. Deficiency in SQSTM1 inhibits the formation of autophagosome and impaired autophagy (54). In Hela cells, SQSTM1 deficiency inhibited the recruitment of MAP1/LC3, resulting in autophagosome formation inhibition (55). Consistently, in our study, we found a close association between SQSTM1 and autophagy through bioinformatic analysis and showed that SQSTM1 knockdown inhibited autophagy induced by Cana in HK2 cells. Therefore, SQSTM1/autophagy may be an important target for Cana treatment.

Furthermore, we explored the mechanism underlying this mode of Cana-mediated SQSTM1/autophagy. m6A is the most prevalent form of posttranscriptional modification in RNA molecules and involved in diverse key biological processes, including alternative splicing, stability, mRNA translation, and miRNA maturation (56–58). Emerging evidence suggests that m6A modification plays a remarkable role in autophagy regulation (59, 60). Herein, Cana treatment markedly increased the m6A level in HK2 cells and mouse kidneys. qPCR analysis further identified that FTO, the first identified m6A demethylase, is the only methyltransferase- or demethylase-related gene with a significant difference. It was reported that FTO plays a role in regulating renal fibrosis, and the m6A modification of lncRNA GAS5 may participate in the process (61, 62). In addition, some studies that have explored the relationship between FTO and autophagy and the results showed that FTO deficiency inhibits mTORC1 signaling and activates autophagy in MEFs (19). Another study has shown that the forced expression of FTO augments the activation of autophagy in 3T3-L1 and porcine primary preadipocytes, suggesting that the role of FTO in autophagy induction is controversial (19). In arsenic-associated human skin lesions, FTO is degraded upon autophagy (63). These results indicate the existence of a probable feedback loop between FTO and autophagy. Our data showed that FTO overexpression resulted in the inhibition of autophagy induced by Cana. Meanwhile, after BafA1 (autophagy inhibitor) treatment, FTO expression increased but failed to restore the downregulation caused by Cana. Thus, FTO may be the upstream of Cana-induced autophagy activation. Overall, a feedback loop may be involved in the FTO and autophagy regulation in tubular cells.

We then explored how FTO regulates autophagy with Cana stimulation. SQSTM1 nuclear mRNA can be modified by m6A reader protein YTHDC1 through methylation in diabetic keratinocytes, and m6A modification may be involved in SQSTM1 expression (64). Consistently, we predicted that SQSTM1 mRNA contained an m6A modification region (position: 1906) with an FTO-binding motif. The results of the RIP-qPCR assay confirmed that FTO interacted with SQSTM1 mRNA. Cana treatment decreased the transcription of FTO, and the m6A methylation of SQSTM1 mRNA increased after Cana treatment. However, FTO overexpression reduced SQSTM1 m6A methylation and suppressed its mRNA stability. These results indicated that Cana increased SQSTM1 mRNA expression *via* FTO. Furthermore, the *in vivo* study employed with SQSTM1 cKO mice confirmed that SQSTM1 in tubular cells played a critical role in the protective effect of Cana on renal fibrosis. Notably, SQSTM1 can contribute to the regulation of fibrotic process *via* the vitamin D receptor or NRF2 pathway, which are independent autophagy programs (65, 66). Thus, the role of SQSTM1 in regulating the protective effects of Cana in renal fibrosis may be either dependent or independent of the autophagy pathways.

**Figure 8:**
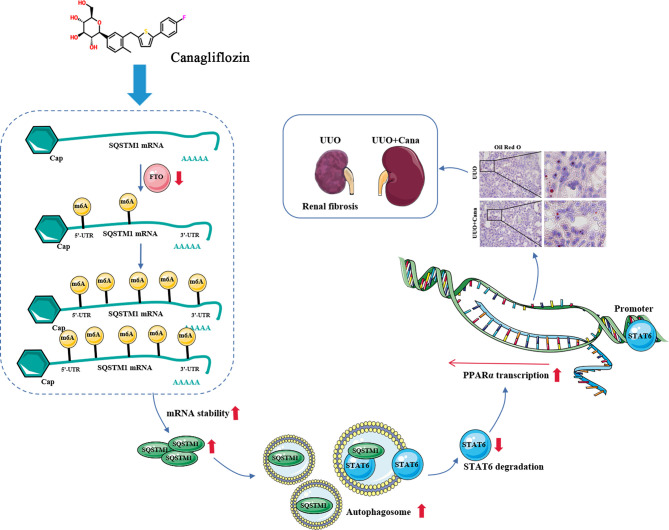
Proposed model for the therapeutic action of Cana against renal fibrosis. Cana attenuates renal tubular cells’ FAO disorder and renal fibrosis by SQSTM1/autophagy-mediated STAT6 degradation in an m6A-dependent manner.

Followed by our previous study that STAT6 activation negatively regulates PPARα signaling and causes FAO disorder and aggravates renal fibrosis (5), here we found that Cana attenuates renal tubular cells’ FAO disorder and renal fibrosis by SQSTM1/autophagy-mediated STAT6 degradation in an m6A-dependent manner. We further clarified that Cana functioned mainly by inhibiting FTO, increasing the stability of SQSTM1 mRNA, and immediately increasing autophagy, which is essential to the degradation of STAT6 (**Figure 8**). Overall, our study will provide valuable insights of Cana into the treatment of chronic kidney disease.

## Data availability statement

The original contributions presented in the study are included in the article/**Supplementary Material**. Further inquiries can be directed to the corresponding authors.

## Ethics statement

The animal study was reviewed and approved by Laboratory Animal Welfare and Ethics Committee of Chongqing University.

## Author contributions

YY and QL designed the experiments and performed part of the experiments. YL and LL performed software, investigation. YM and LX provided technical and material support. GL and YD performed and analyzed the result of mouse model. ST and JL performed conceptualization, visualization, writing—review and editing, supervision, and project administration. All authors contributed to the article and approved the submitted version.
