# Supplementary material for: m6A eraser FTO modulates autophagy by targeting SQSTM1/P62 in the prevention of canagliflozin against renal fibrosis
Source: Front Immunol. 2023 Jan 4;13:1094556. doi: 10.3389/fimmu.2022.1094556 (PMC9845768; doi:10.3389/fimmu.2022.1094556)

Supplementary Information

Contents

Supplementary Figure S1

Supplementary Figure S2

Supplementary Figure S3

Supplementary Figure S4

Supplementary Figure S5

Supplementary primer list

Supplementary figure legends

Figure S1. Cana was harmless to mice renal function. (**A**) The levels of BUN in kidney. (B) Serum lipid from the indicated group were assayed.

Figure S2. Cana was innoxious to kidney in WT *Atg7*^{cKO} mice. Mice received intragastric administration of saline or Cana (20 mg/kg). (**A**) Representative microphages for H&E and Sirius Red staining of kidney sections.

Figure S3. The effect of FTO on autophagy. (A) HK2 cells were transfected with a tandem mRFP-GFP-LC3 construct for 24 h and then received the indicated treatments. Representative micrographs of the indicated cells were shown.

Figure S4. SQSTM1 mRNA was predicted as the target of FTO. (**A**) Online predictive tool (SRAMP, http://www.cuilab.cn/sramp) revealed the potential m6A methylation sites with high probability. (**B**) AUGGACU were the binding sites or motif of FTO. (**C**) The potential m6A methylation sites of SQSTM1 mRNA were identified with very high confidence.

Figure S5. Cana was harmless to WT or renal tubular SQSTM1 deletion mice. (**A**) Representative microphages for H&E and Sirius Red staining of kidney sections with indicated treatments.

Supplementary primer list

Table 1

Primer Sequence (5’-3’)		
h-FN	Forward	TGGCTGTCAGTCAAAGCAAG
	Reverse	CTCGGCTTCCTCCATAACAA
h-α-SMA	Forward	CAGCCAAGCACTGTCAGG
	Reverse	CCAGAGCCATTGTCACACAC
h-TGFβ	Forward	GTACCTGAACCCGTGTTGCT
	Reverse	GTATCGCCAGGAATTGTTGC
h-METTL3	Forward	TTGTCTCCAACCTTCCGTAGT
	Reverse	CCAGATCAGAGAGGTGGTGTAG
h-METTL14	Forward	CTGAGAGTGCGGATAGCATTG
	Reverse	GAGCAGATGTATCATAGGAAGCC
h-FTO	Forward	ACTTGGCTCCCTTATCTGACC
	Reverse	TGTGCAGTGTGAGAAAGGCTT
h-ALKBH5	Forward	CGGCGAAGGCTACACTTACG
	Reverse	CCACCAGCTTTTGGATCACCA
h-WTAP	Forward	CTTCCCAAGAAGGTTCGATTGA
	Reverse	TCAGACTCTCTTAGGCCAGTTAC
h- SREBP-1c	Forward	TGCATTTTCTGACACGCTTC
	Reverse	CCAAGCTGTACAGGCTCTCC
h- SREBP-2	Forward	CCGCCTGTTCCGATGTACAC

	Reverse	TGCACATTCAGCCAGGTTCA
h- FASN	Forward	GGAAGCTGCCAGAGTCGGAGAACT
	Reverse	TGAGGGTCCATCGTGTGTGCCT
h- PPARα	Forward	ATGGTGGACACGGAAAGCC
	Reverse	CGATGGATTGCGAAATCTCTTGG
h- ACOX-1	Forward	CCAAGCTTTCCTGCTCAGTGTT
	Reverse	CCCCCAGTCCCTTTTCTTCA
h-GAPDH	Forward	CTGACTTCAACAGCGACACC
	Reverse	TGCTGTAGCCAAATTCGTTGT
h-CPT-1α	Forward	TCGTCACCTCTTCTGCCTTT
	Reverse	ACACACCATAGCCGTCATCA
h-CD36	Forward	GGCTTAATGAGACTGGGACCA
	Reverse	TCACCACACCAACACTGAGT
h-FATP1	Forward	TGACGTGCTCTATGACTGCC
	Reverse	ACTTGATGCAGTCGTCCCAG
h-APOA4	Forward	GGTGACCTGCAGAAGAAGCT
	Reverse	CCAGCTCCTTCCCAATCTCC
h-MTTP	Forward	ATACCTGCAGCCTGACAACC
	Reverse	TTCTTCGCAGTCCTGAGGTG
h-STAT6	Forward	CACCGAGGGAATGGCGCACCGTTTG
	Reverse	AAACCAAACGGTGCGCCATTCCCTC
h-Arginase1	Forward	GGCTGGTCTGCTTGAGAAAC

	Reverse	TTCCCACAGACCTTGGATTC
m-β-actin	Forward	AAGGCCAACCGTGAAAAGAT
	Reverse	GTGGTACGACCAGAGGCATAC
m-FN	Forward	CATGAAGGGGGTCAGTCCTA
	Reverse	TAGGTTTGCAGGTCCATTCC
m-α-SMA	Forward	GTCCCAGACATCAGGGAGTAA
	Reverse	TCGGATACTTCAGCGTCAGGA
m-TGF-β1	Forward	GACTCTCCACCTGCAAGACC
	Reverse	GACTGGCGAGCCTTAGTTTG
m-METTL14	Forward	CTGAGAGTGCGGATAGCATTG
	Reverse	GAGCAGATGTATCATAGGAAGCC
m-FTO	Forward	TTCATGCTGGATGACCTCAATG
	Reverse	GCCAACTGACAGCGTTCTAAG
m-ALKBH5	Forward	CGCGGTCATCAACGACTACC
	Reverse	ATGGGCTTGAACTGGAACTTG
m-WTAP	Forward	GAACCTCTTCCTAAAAGGTCCG
	Reverse	TTAACTCATCCCGTGCCATAAC
m- SREBP-1c	Forward	GGGCAAGTACACAGGAGGAC
	Reverse	AGATCTCTGCCAGTGTTGCC
m- SREBP-2	Forward	CAGGCGACCAGGAAGAAGAG
	Reverse	CGGAACTGCTGGAGAATGGT
m- FASN	Forward	GACCTCAGGCTGCAGTGAAT

	Reverse	CACCTTCTTGAGAGCCTGCA
m- PPARα	Forward	AGTGCCCTGAACATCGAGTG
	Reverse	TTGCAGCTCCGATCACACTT
m- ACOX-1	Forward	GGGGAACATCATCACAGGGG
	Reverse	ATCATAGCGGCCGAGAACAG
m-CPT-1α	Forward	TTGGACGAATCGGAACAGGG
	Reverse	CCATGCAGCAGAGATTTGGC
m-CD36	Forward	ATTGTACCTGGGAGTTGGCG
	Reverse	CAGCCAGGACTGCACCAATA
m-FATP1	Forward	CGCCGATGTGCTCTATGACT
	Reverse	ACACAGTCATCCCAGAAGCG
m-APOA4	Forward	GGAGCACCTGAAGCCCTATG
	Reverse	CATCATCGAGGTGTGCAGGT
m-MTTP	Forward	AATGCGGGTCAACAGAGAGG
	Reverse	CCCCGGACCAGATGAAGAAG
m-STAT6	Forward	GATGACTGTGGAAAGGGACCA
	Reverse	GGATGGACTGTGGAGGATACC
m-Arginase1	Forward	CGCCTTTCTCAAAAGGACAG
	Reverse	TTTTTCCAGCAGACCAGCTT

Figure S1

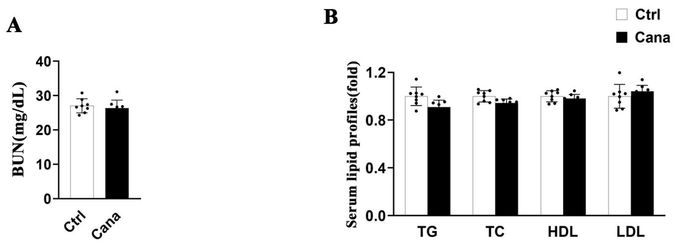
A
B
Ctrl
Cana
BUN(mg/dL)
40
30
20
10
0
Ctrl
Cana
Serum lipid profiles(fold)
1.2
0.8
0.4
0.0
TG
TC
HDL
LDL

Figure S2

A

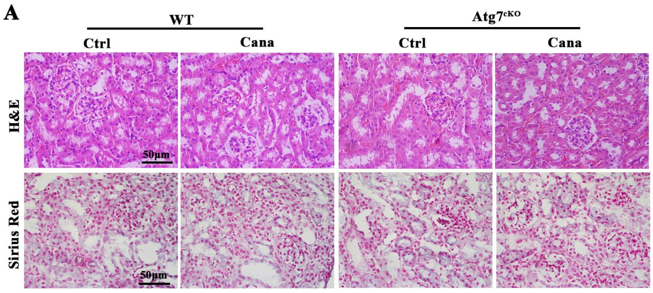

Figure S3

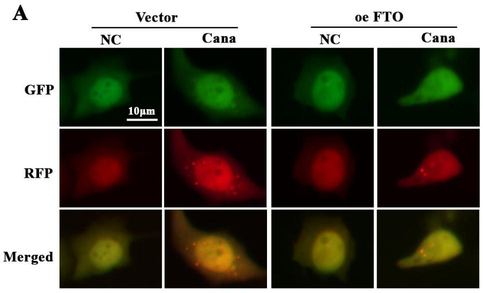

Figure S4

A

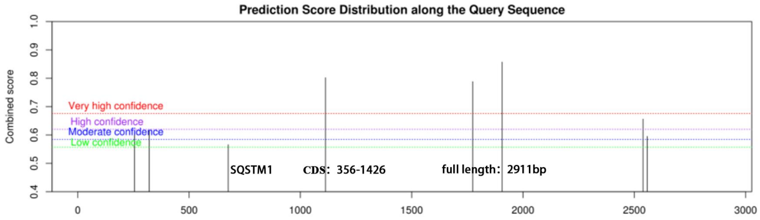

B

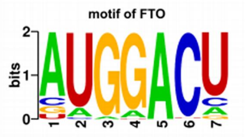

C

#	Position	Sequence context	Structural context	Local structure visualization	Score(binary)	Score(knn)	Score(spectrum)	Score(combined)	Decision
1	255	CAAGA ACCUG GCUUU<br>UAGCU GAACU AAGGA<br>GAAAG UCCUA CAACA	N/A	N/A	0.645	0.51	0.577	0.611	m^6A site (**Moderate** confidence)
2	321	UUGAG AAAGG AUGAG<br>GACGG GGACU UGGUU<br>GCCUU UUCCA GUGAC	N/A	N/A	0.677	0.669	0.529	0.617	m^6A site (**Moderate** confidence)
3	676	GGCUC CGGAA GGUGA<br>AACAC GGACA CUUCG<br>GGUGG CCAGG AUGGG	N/A	N/A	0.583	0.741	0.522	0.566	m^6A site (**Low** confidence)
4	1113	AACUG UUCAG GAGGA<br>GAUGA UGACU GGACC<br>CAUCU GUCUU CAAAA	N/A	N/A	0.827	0.450	0.813	0.802	m^6A site (**Very high** confidence)
5	1774	ACUUU CUCUU UUGUU<br>UUAAA UGACU CAUAG<br>GUCCC UGACA UUUAG	N/A	N/A	0.829	0.449	0.775	0.788	m^6A site (**Very high** confidence)
6	1906	GUGUU GUGGG CUUCC<br>UGCUG GGACU GAGAA<br>GGCUC ACGAA GGGCA	N/A	N/A	0.896	0.784	0.813	0.857	m^6A site (**Very high** confidence)
7	2539	GCUGC UCACU GUCCA<br>CAUGU GAACU UUUUC<br>UAGGU GGCAG GACAA	N/A	N/A	0.728	0.583	0.565	0.656	m^6A site (**High** confidence)
8	2558	UGAAC UUUUU CUAGG<br>UGGCA GGACA AAUUG<br>CGCCC AUUUA GAGGA	N/A	N/A	0.592	0.406	0.624	0.595	m^6A site (**Moderate** confidence)

Figure S5

A

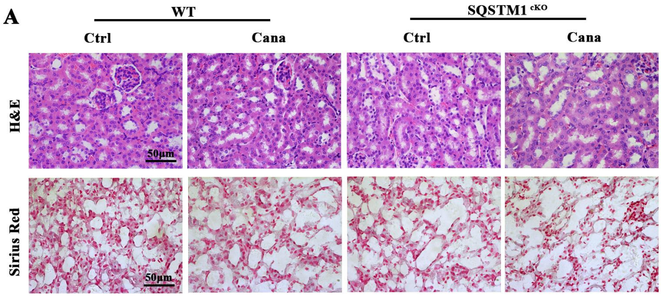

Supplement: Supplementary file 1 [file DataSheet_1.pdf]
